# Bacterial Diversity in Aquacultured African Catfish and Source Pond Water in Buea, Cameroon

**DOI:** 10.1155/ijm/6132661

**Published:** 2025-05-19

**Authors:** Gordon Takop Nchanji, Bertrand Tatsinkou Fossi, Jerome Fru-Cho, Robert Adamu Shey, Akeson Akeh Andoh, Andrielle L. Kemajou Tchamba, Nur A. Hasan, Samuel Wanji

**Affiliations:** ^1^Department of Microbiology and Parasitology, University of Buea, Buea, Cameroon; ^2^Tropical Disease Interventions, Diagnostics, Vaccines and Therapeutics (TroDDIVaT) Initiative, Buea, Cameroon; ^3^Research Foundation for Tropical Diseases and the Environment (REFOTDE), Buea, Cameroon; ^4^Department of Biochemistry and Molecular Biology, University of Buea, Buea, Cameroon; ^5^Department of Fisheries and Aquatic Resources Management, Faculty of Agriculture and Veterinary Medicine, University of Buea, Buea, Cameroon; ^6^Department of Biology, University of Mississippi, Oxford, Mississippi, USA; ^7^EzBiome Inc., Gaithersburg, Maryland, USA

**Keywords:** African catfish, aquaculture, bacteriome, *Clarias gariepinus*, microbiome, sequencing

## Abstract

The catfish is a prominent freshwater fish species farmed in Cameroon to meet the escalating demand for fish products. Despite considerable growth potential, there are concerns about the occurrence of bacteria pathogenic to both fish and humans within aquaculture systems. Research on the microbiome of catfish and their habitats remains largely unexplored. Given the critical importance of understanding the microbial composition within aquaculture systems to ensure food safety and protect public health, this study aimed to generate vital preliminary data by investigating the bacteriome of catfish gills and intestines and pond water environment in Cameroon using 16S rRNA gene amplicon sequencing. The findings revealed a diverse bacterial community (30 phyla, 678 genera, and 1056 species), with Fusobacteria, Bacteroidetes, Proteobacteria, Firmicutes, and Verrucomicrobia collectively representing over 93% of the bacterial community observed. Notably, Fusobacteria emerged as the dominant phylum in catfish gills (49.98%) and intestines (65.3%), while Proteobacteria predominated in the pond water environment (40.24%). Bacteria of genus *Cetobacterium* dominated all three samples (gills, 49.93%; intestines, 65.19%; and pond water, 23.85%). Furthermore, this study identified many bacterial genera, including potential fish pathogens such as *Edwardsiella*, *Aeromonas*, *Plesiomonas*, and *Flavobacterium*, and human gut bacteria such as *Clostridium* and *Bacteroides*, alongside potential beneficial probiotic bacteria such as *Lactococcus* spp. The coexistence of both potentially pathogenic and probiotic species underscores ecological complex dynamics within freshwater fish aquaculture and highlights the need for thorough microbial management strategies. This study provides insights into the bacterial landscape of Cameroonian aquaculture, revealing potential risks and benefits of catfish farming.

## 1. Introduction

Catfish ranks among the most cultivated freshwater fish species in Cameroon, where aquaculture as an emerging practice holds great potential to bridge the huge gap that exists between fish demand and local production. The high demand for fish imposes a significant economic burden on the country, resulting in large-scale importation of frozen fish. In 2019 alone, the Cameroonian government imported around 185,000 tonnes of fish, costing an estimated 294 million USD [1]. To alleviate the significant economic burden associated with the importation of frozen fish, the Cameroonian government has prioritized the development of local aquaculture to bolster local fish output. Even though aquaculture is an expensive economic activity (due to initial high startup costs including the high cost of fish feeds, fingerlings, and land acquisition) [2], it has proven to be a lucrative venture in Cameroon [3].

The aquaculture production model is the most rapidly growing animal production sector, currently accounting for approximately 50% of global fish production for food [4]. This rapid transition has been driven by the realization that the capture fisheries model was unsustainable due to rapidly increasing demand, which led to overfishing, increased marine pollution, and global climate change [5]. While the practice of intensive freshwater fish aquaculture addresses the huge demand, it is characterized by several risk factors that lead to microbial contamination of fish [6, 7]. The manipulation of breeding cycles, high stocking density of fish in the pond, and the feeding or fertilization of ponds with agricultural by-products, including animal droppings, impose a certain amount of stress on the fish, thus increasing the predisposition of fish to microbial contamination.

Bacteria (*Aeromonas hydrophila*, *Bacillus* spp., and *Vibrio cholerae*), viruses (tilapia lake virus), parasites (like Myxobolus and Henneguya), and fungi (*Aspergilllus* spp. and *Trichosporon* spp.) are among the common contaminants found in fish, often originating from the aquatic environment [8–10]. Given numerous reports on the occurrence of pathogenic bacterial species in aquaculture fish [10–15] and humans [10, 16, 17], it is critical to deploy precautionary measures to enhance disease control and prevention. The use of antibiotics is one of such method, and there is no discrimination between the classes of therapeutic antimicrobials used in humans and food-producing animals [18]. It has been reported that approximately 70%–80% of antibiotics fed to fish are excreted and persist in the aquaculture environment as fish are unable to effectively metabolize these antibiotics [19, 20]. As a consequence, these residual unmetabolized antibiotics exert selective pressure on the microbiota of aquaculture ecosystems [21, 22].

Infectious disease remains one of the major challenges for the aquaculture industry [23, 24], leading to high mortality rates in fish and substantial economic losses for fish farmers. The problem of the disease in the aquaculture setting is further worsened by antibiotic resistance wherein resistance patterns observed in inland animal husbandry have also been observed in aquaculture [25]. The widespread dissemination of resistant organisms and genes across humans, farmed animals, fish, and the environment underscores the threat posed by antimicrobial resistance (AMR) to human and animal health [10], highlighting the urgent need for the controlled use of antibiotics in animal husbandry and aquaculture to preserve available antimicrobials [26].

Fish vaccination and breeding for disease resistance have shown great promise for fish health management, but a sustainable solution also should involve continuous disease monitoring and surveillance, rapid diagnosis, and strengthened biosecurity in hatcheries and breeding centers [27]. Detailed knowledge of the organisms circulating within aquaculture setups will enable better management of fish farms and serve as a baseline for evaluating interventions aimed at improving output, and most importantly, ensuring the health and safety of fish products for human consumption. With the continuous rise and spread of antibiotic resistance and knowledge of the strong influence of gut microbiota on fish health, information on the dominant populations in the fish gut can provide a better insight into obtaining good bacterial species that can serve as probiotic candidates for feed formulations that can contribute to improving fish health, nutrient uptake, and disease resistance within the sub-Saharan African (SSA) aquaculture ecosystem [28]. Moreover, knowledge of human pathogenic genera circulating within aquaculture systems can help identify hazards or potential risks for both the manipulating and consuming human population.

Few studies, with none reported in SSA, have explored the bacteriome of the African catfish, *Clarias gariepinus* [29], and microbiome investigations in other fish species have focused primarily on factors influencing their growth, survival, and yield. However, there is a notable gap in such detailed studies within the SSA context. Given the importance of locally generated data for the regional aquaculture industry, this study focused on generating preliminary data by characterizing the microbiome of aquacultured African catfish gills, intestines, and the surrounding pond water environments.

## 2. Methods

### 2.1. Collection of Samples

**Figure 1:**
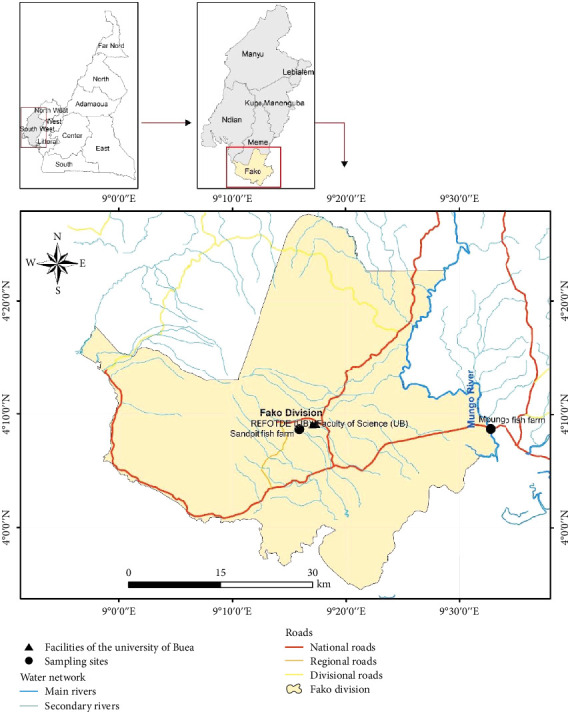
Map of study site depicting country, region, and arrow pointing to sampling site.

Catfish and pond water samples were purposively collected from the main commercial fish farm in Sandpit-Buea (Figure 1) which serves Buea's community with table catfish. Samples were collected from a concrete pond, rectangular in shape, and adequately covered by roofing sheets, with access to the farm restricted from the general population. The feed was supplemented with antibiotics (oxytetracycline is spread on fish feed at the rate of 50 g/10 kg), and fish were reared in a flow-through system that uses protected underground clean spring water. Samples were collected between January and March of 2023 in two visits. Then, 10 fish samples comprising five females and five males were caught using a scoop net, while pond water was collected in four sterile 50-mL tubes as water was emptied through an escape nozzle in preparation for fishing. Fish samples were transported in large open-mouth transparent disinfected buckets containing fish pond water, while water samples were transported on icepacks in small cooler flasks to the laboratory within 1 h of collection. Water quality analysis was not performed.

### 2.2. Sample Processing and Total DNA Extraction

Upon arrival at the laboratory, fish were euthanized by decapitation. The skin of each fish was passively washed by pouring sterile water over the skin surface of the fish before processing. Prior to dissection, the skin surface of the fish was cleaned with 70% alcohol, and 1 g of the gills and intestines from individual fish were separately dissected out and ground in a disinfected mortar. Samples from five individual fishes of the same gender were pooled to form a single sample. Five samples, including separate pools of gills and intestines from both sexes and pooled source water samples, were processed for extraction during each field visit. Subsequently, 250 mg of each homogenous ground subsample of gills and intestines were transferred to a ZR BashingBead lysis tube (0.1 and 0.5 mm) and 750-*μ*L ZymoBIOMICS lysis solution was added to the tube for processing as per the optimized manufacturer's instructions (ZymoBIOMICS DNA Miniprep Kit_D4300). Water samples were centrifuged, and pellets were pooled. Then, 250 *μ*L of each homogenous pooled pellet from the water sample was transferred to a ZR BashingBead Lysis Tube (0.1 and 0.5 mm), and 750-*μ*L ZymoBIOMICS lysis solution was added to mix in the tube. DNA extraction was conducted according to the manufacturer's instructions and included a negative control of sterile water. The concentration of the resulting genomic deoxyribonucleic acid (gDNA) samples was measured using a Qubit fluorometer (Thermo Fisher Scientific, United States), following the manufacturer's instruction. All DNA samples were stored at −20°C until shipping to EzBiome Inc. (Gaithersburg, MD, United States) for amplicon sequencing and bioinformatics analysis.

### 2.3. 16S rRNA Gene Amplicon Sequencing

Upon receipt of samples, gDNA concentration was further validated on a Qubit 4.0 Fluorometer using Qubit dsDNA High Sensitivity Assay Kit (ThermoFisher Scientific, Waltham, MA, United States). This procedure was followed by 16S rRNA gene amplicon sequencing using methods optimized by EzBiome Inc. (Gaithersburg, MD, United States). The 16S rRNA V3-V4 regions within the ribosomal transcript were amplified using the primer pair (Illumina-F: TCGTCGGCAGCGTCAGATGTGTATAAGAGACAGCCTACGGGNGGCWGCAG and Illumina-R: GTCTCGTGGGCTCGGAGATGTGTATAAGAGACAGGACTACHVGGGTATCTAATCC) which contains the gene-specific sequences and Illumina adapter overhang nucleotide sequences. Amplicon polymerase chain reaction (PCR) was performed to amplify the template out of input DNA samples as previously described [30]. Each 25 *μ*L of PCR contained 12.5 ng of sample DNA as input, 12.5 *μ*L 2x KAPA HiFi HotStart ReadyMix (Kapa Biosystems, Wilmington, MA), and 5 *μ*L of each primer (1 *μ*M). PCRs were carried out using the following protocol: an initial denaturation step at 95°C for 3 min followed by 25 cycles of denaturation (95°C, 30 s), annealing (55°C, 30 s), and extension (72°C, 30 s), and a final elongation for 5 min at 72°C. PCR products were cleaned up from the reaction mix with Mag-Bind RxnPure Plus magnetic beads (Omega Bio-tek, Norcross, GA). A second index PCR amplification, used to incorporate barcodes and sequencing adapters into the final PCR product, was performed in 25-*μ*L reactions, using the same master mix conditions as described above. Cycling conditions were as follows: 95°C for 3 min, followed by 8 cycles of 95°C for 30⁣^″^, 55°C for 30⁣^″^, and 72°C for 30⁣^″^. A final, 5-min elongation step was performed at 72°C. The libraries were then normalized and pooled. The pooled library was checked using an Agilent 2200 TapeStation and sequenced on the MiSeq (Illumina, San Diego, CA) on a 500-cycle (2 × 250 bp paired-end) run.

### 2.4. Bioinformatic Analysis

The bioinformatics analysis included amplicon taxonomic assignment and comparative statistical analyses [31]. The EzBioCloud microbiome taxonomy profiling platform (http://www.ezbiocloud.net) was used to perform taxonomic profiling of 16S sequencing paired-end reads as described by Yoon et al. [32]. EzBioCloud filtered out sequences of low quality by read length (< 438 bp or > 468 bp) and averaged *Q* values less than 25, while the DUDE-Seq software was used to denoise and extract non-redundant reads. To eliminate chimera sequencing, the UCHIME algorithm was applied against the EzBioCloud 16S chimera-free database. The USEARCH program was used to calculate sequence similarities of the query single-end reads for taxonomic assignment against the EzBioCloud 16S database. Using a cutoff set at 97% sequence similarity, the UPARSE algorithm was used to cluster sequencing reads into operational taxonomic units (OTUs) while the UCLUST tool was used to cluster each sample reads into many.

To estimate the functional profiles of the microbiome identified using 16S rRNA gene amplicon sequencing, the EzBioCloud 16S-based microbiome taxonomic profile (MTP) pipeline used the PICRUST algorithm. The KEGG (Kyoto Encyclopedia of Genes and Genomes) orthology and pathway database were used to bioinformatically annotate the functional abundance profiles of the microbiome wherein the vector of gene counts for each OTU was multiplied by the abundance of that OTU in each sample. The subsampling, generation of taxonomy plots/tables and rarefaction curves, and calculation of species richness, coverage, and alpha and beta diversity indices were performed in the EzBioCloud App [32]. Abundance-based coverage estimator (ACE), Chao1, Jakknife, and the number of OTUs found in the MTP index were used to measure microbial richness. Using the Wilcoxon rank-sum test, the diversity for each group was estimated using the Shannon, Simpson, and Phylogenetic *α*-diversity indices. The Bray–Curtis dissimilarity distances based on the taxonomic abundance profiles were used to calculate the beta diversity. The statistical significance of *β*-diversity was measured using permutational multivariate analysis of variance (PERMANOVA). Principal component analysis (PCoA) was used to cluster different groups based on the abundance Jaccard distance metric.

## 3. Results

### 3.1. Bacteriome Profiles

Nine out of the 10 DNA samples underwent successful PCR amplification of the targeted V3–V4 regions of the 16S rRNA gene, followed by adequate sequencing data generation for microbiome analysis. From the nine samples, representing three sample types, that is, gills, intestines, and pond water, a total of 162,687 valid reads were obtained, ranging from 10,134 to 26,433 reads per sample. All valid reads underwent taxonomic assignment through clustering, using a sequence similarity threshold of 97%. The mean number of valid reads that were identified at the species level in gills, intestines, and pond water were 11,152, 16,091, and 16,616, respectively. The bacteriome profile of all sample types' analysis revealed an incredibly rich and taxonomically diverse bacterial community, comprising 30 bacterial phyla, 72 classes, 144 orders, 308 families, 678 genera, and 1056 species across samples.

#### 3.1.1. Bacteriome Profile at Phylum Level

**Figure 2:**
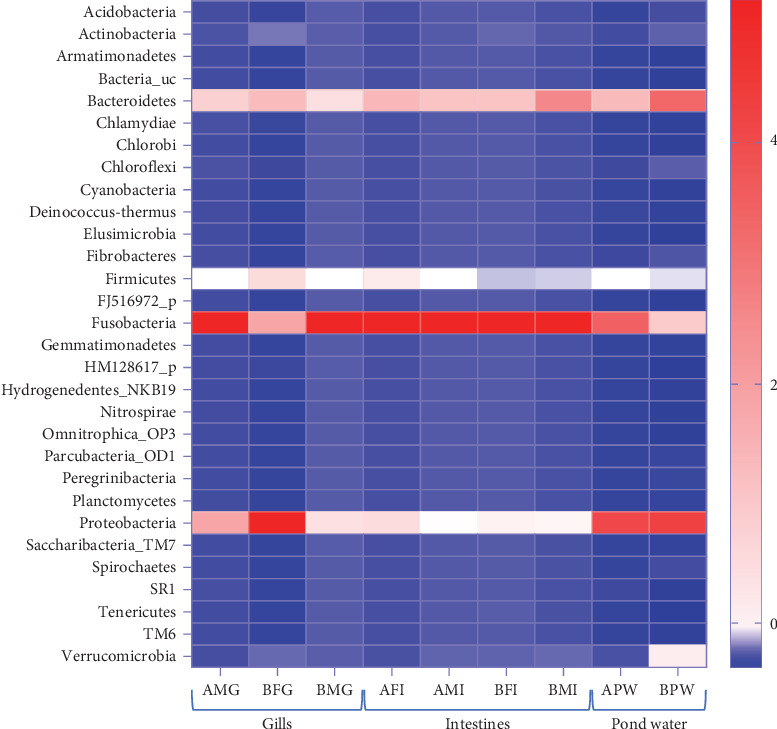
Heatmap plot of the relative abundance (*z*-score standardised) of various phyla revealed in bacteriome of catfish gills (*n* = 3), catfish intestines (*n* = 4), and pond water (*n* = 2) (A: first collection; B: second collection; M: male; F: female; G: gills; I: intestines; PW: pond water).

**Figure 3:**
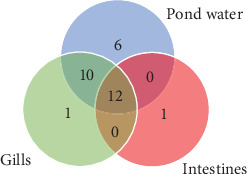
Venn diagram representing shared operational taxonomic units (OTUs) of phyla between the gills, intestines, and pond water.

In the taxonomy assignment at the phylum level across the three sample types, a total of 30 phyla were identified (Figure 2). Among these, 12 phyla (Fusobacteria, Bacteroidetes, Proteobacteria, Firmicutes, Verrucomicrobia, Actinobacteria, Chloroflexi, Saccharibacteria_TM7, Spirochaetes, Cyanobacteria, Planctomycetes, and Acidobacteria) were found to be shared among all three sample types, while 10 phyla were shared specifically between the gills and pond water bacteriome (Figure 3). The gill and intestinal samples each exhibited one distinct phylum, while pond water samples revealed six unique phyla.

**Figure 4:**
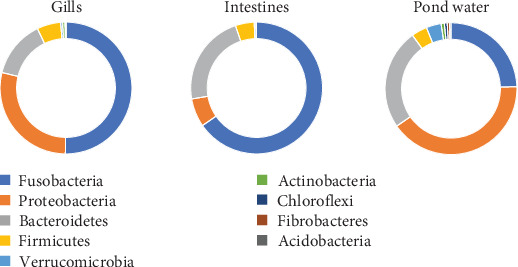
Donut presentation of mean percent abundance of phyla by sample category. Different colors represent different phyla within each category.

Among the 12 shared phyla, Fusobacteria, Bacteroidetes, Proteobacteria, Firmicutes, and Verrucomicrobia were predominant, with each representing over a 3% mean percent abundance in at least one sample type and comprising over 93% of all identified phyla (Figure 4). The percentage abundance of Proteobacteria was higher in pond water (40.24%) compared with catfish gills and intestines (28.51% and 6.98%, respectively). Bacteroidetes had a mean percent abundance of 22.44% and 24.28% in intestines and pond water samples, respectively, with the lowest abundance observed in gill samples (14.03%). The mean percent abundance of Fusobacteria, Firmicutes, and Verrucomicrobia were not significantly different (*p* < 0.05) across sample types. Fusobacteria exhibited slightly higher mean percent abundance in intestines (65.3%) and gills (49.98%) and lowest in pond water (24.12%). Firmicutes showed slightly higher abundance in the gills and intestines, while Verrucomicrobia was slightly more abundant in pond water samples. The contribution of all other reported phyla summed up to approximately 6.3% of all identified phyla.

#### 3.1.2. Bacteriome Profile at Genus Level

**Figure 5:**
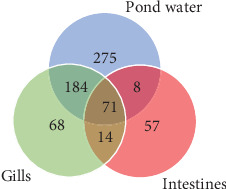
Venn diagram representing shared operational taxonomic units (OTUs) of genera between sample types.

**Figure 6:**
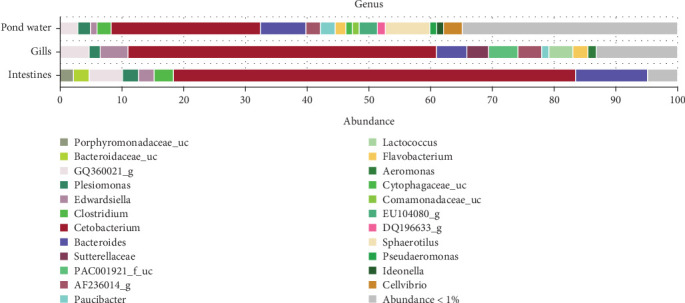
Mean percent abundance of genera by sample category. Different colors represent different genera within each category.

At the genus level, taxonomic assignment revealed a total of 678 genera across the three sample types.  Figure 5 provides an overview of how these genera are shared among the samples. Specifically, 71 genera were shared among gills, intestines, and pond water, while 184 genera were shared among pond water and gill samples. Pond water contained the highest proportion of distinct genera identified, with a total of 275 distinct genera. Overall, the most abundant genera identified were *Cetobacterium,* found in all three sample types with a mean abundance of 49.93%, 65.19%, and 23.85% in the gills, intestines, and pond water samples (Figure 6), respectively.

Figure 6 illustrates the mean abundance of the most abundant genera identified. Within intestinal samples, OTUs of the genera Porphyromonadaceae_uc (2.19%) and Bacteroidaceae_uc (2.54%) were slightly more abundant, whereas *Lactococcus* (3.83%), *Aeromonas* (1.46%), PAC001921_f_uc (4.7%; class: Betaproteobacteria) and Sutterellaceae_uc (3.56%) were more abundant in the gill samples. Pond water samples exhibited eight genera with a mean abundance greater than 1%, compared to less than 1 in the other samples. These included *Sphaerotilus* (7.06%), *Cellvibrio* (3.05%), EU104080_g (2.91%), DQ196633_g (1.3%), *Pseudaeromonas* (1.12%), Comamonadaceae_uc (1.07%), Cytophagaceae_uc (1.06%) and Ideonella (1.05%). *Paucibacter* (gills: 1.18%; pond water 2.36%), AF236014_g (gills: 3.83%; pond water 2.33%) and *Flavobacterium* (gills: 2.48%; pond water 1.64%) genera were relatively more abundant in gills and pond water samples, while *Clostridium* was relatively more abundant in intestines (3.08%) and pond water (2.21%) samples.

### 3.2. Shared and Unique Bacteriome Taxa of Catfish and Pond Water Samples

Table 1 shows bacterial genera accounting for ≥ 1% of sequences within each sample type. Shared taxa are present in all three sample types. Bacterial genera shared across fish and pond water included *Cetobacterium*, the most prevalent bacterial genera, and the fish pathogenic genera *Edwardsiella, Aeromonas, Plesiomonas*, and *Flavobacterium*. Shared bacteria also included human gut bacteria of the genus *Clostridium* and *Bacteroides*, as well as bacteria of the probiotic genera of *Lactococcus*. Bacteria of the genus *Sphaerotilus, Cellvibrio,* and *Paucibacter* were shared among the gills and pond water, though more abundant in pond water. Sutterellaceae_uc and PAC001921_f_uc OTUs, which belong to bacteria of the phylum proteobacteria, were found exclusively in the gills.

### 3.3. Diversity Analysis

**Figure 7:**
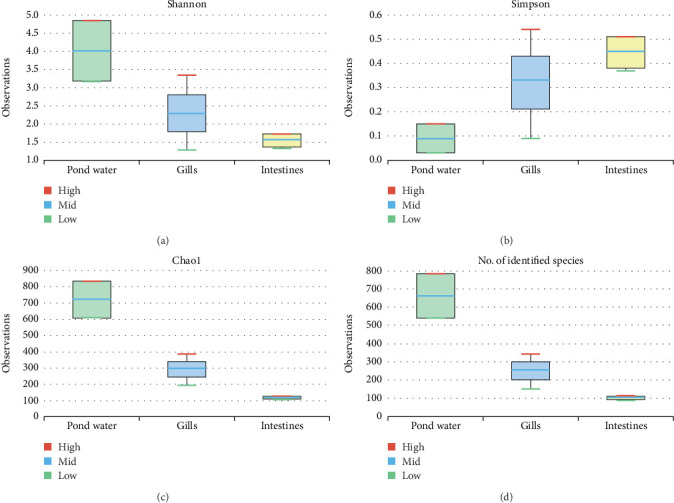
Alpha diversities of the bacteriome of catfish gills, intestines and pond water as calculated by (a) Shannon index, (b) Simpson, (c) Chao1, and (d) observed species.

**Figure 8:**
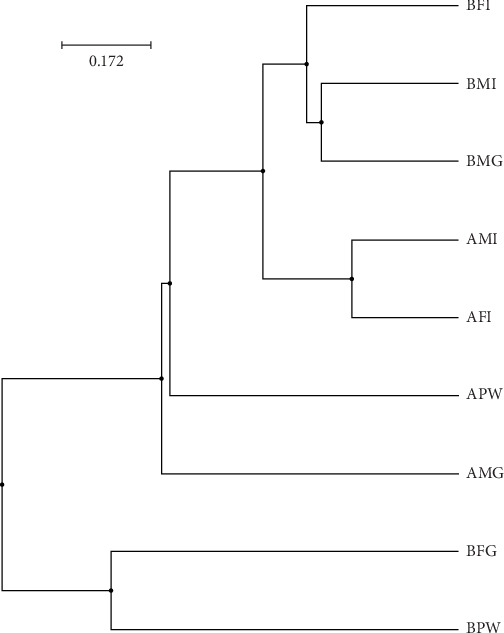
Beta diversity of the bacteriome of catfish gills, intestines, and pond water. UPGMA cluster based on Bray–Curtis dissimilarity index (A: first collection; B: second collection; M: male; F: female; G: gills; I: intestines; PW: pond water).

The alpha diversity assessment of the three sample types was performed using Shannon, Simpson, Chao1, and observed species diversity indices. These indices provide insights into species richness, evenness, and community composition. These revealed that microbial diversity was highest in pond water as compared to gills and intestines, with the lowest diversity observed in intestines. Chao1 (species richness) and number of OTUs (Figure 7) demonstrated the significant differences of alpha diversity between gills and intestinal samples following Wilcoxon rank-sum test (*p* value = 0.034). Thus, the alpha diversity of the gills and intestines is different. The beta diversity indices as presented by the Bray–Curtis dissimilarity index showed that the intestinal samples clustered together separately from other samples of the two types (Figure 8). PERMANOVA analysis showed a significant difference between gills and intestinal samples (*p* value = 0.033 and *q* value = 0.084).

### 3.4. Functional Abundance of Kyoto Encyclopedia of Genes and Genomes Orthologies (KOs)

The most abundant KOs annotated from taxonomic data were significantly different among the sample types (Table 2). In fact, analysis revealed that the most abundant KOs were from the microbiome of the intestines with K21572 (0.495) that encodes for the starch-binding outer membrane protein, SusD/RagB family, and K02014 that encodes for the iron complex outer membrane receptor protein (0.427) significantly highly expressed in intestines as compared to pond water and gill microbiome. KOs, which were significantly highest in pond water, included K00059 (0.306) that encodes for the 3-oxoacyl-[acyl-carrier protein] reductase and K03392 (0.394) that encodes for the aminocarboxymuconate-semialdehyde decarboxylase.

## 4. Discussion

The importance of fish microbiota on fish health has been widely recognized in various studies. Dominant bacterial species within the gut microbiota are particularly considered strong candidates for fish probiotics, especially in fish aquaculture settings. Despite the significance of catfish aquaculture in Cameroon and the broader sub-Saharan region, microbiome studies characterizing microbial communities associated with catfish and related environments are limited, with none reported in Cameroon or within the sub-Saharan region. Given the scale of catfish aquaculture in the country and on the continent, there is a notable gap in understanding the microbiome associated with these fish species, their differences, and potential role in fish health. This microbiome study represents a preliminary attempt to investigate the bacteriome associated with the gills and intestines of the African catfish *Clarias gariepinus*, as well as its pond water aquaculture in Cameroon. By shedding light on the microbial communities inhabiting these crucial environments, the study aims to provide useful insights to drive understanding of the microbial factors influencing catfish health as well as potentially contribute to the development of catfish aquaculture-oriented probiotics in the region.

Analyzing the bacterial taxa present in fish gills and intestines, the prevalence and abundance of Fusobacteria across all sample types, with *Cetobacterium* emerging as the most dominant genus, aligns with findings from previous studies. Indeed, research by [33–36] has consistently reported *Cetobacterium* as a major component of freshwater fish gut microbiota. This observation holds for other freshwater fish species, including tilapia [37, 38], as well as more recently for the African catfish, as indicated by Skvortsova et al. [28]. These findings suggest that *Cetobacterium* may indeed be a common resident of the bacterial microbiota in catfish and their environment, playing a significant role in catfish biology. The widespread presence of *Cetobacterium* across different freshwater fish species [34, 35] implies its importance as a key component of the fish gut microbiota in these aquatic environments. This supports the notion that globally, *Cetobacterium* could be considered a characteristic species within the guts of freshwater fishes, highlighting its potential significance in understanding fish health and ecology.

The capability of *Cetobacterium* species to produce cobalamin (Vitamin B_12_), along with antimicrobial metabolites, underscores their potential significance as functional symbionts with freshwater fish guts, including those of the African catfish *C. gariepinus*. Previous studies have demonstrated that Vitamin B_12_ produced by *Cetobacterium somerae* can enhance host resistance against pathogen infection by promoting beneficial interactions within the gut microbiota [39, 40]. Given these properties, *Cetobacterium* could indeed serve as an important functional symbiont [36] in freshwater fish guts, including the African catfish *C. gariepinus*. Harnessing the presence of *Cetobacterium* as a core member of the catfish bacteriome is a promising prospect for the development and testing of tailored feeds suitable for local species or specific aquaculture practices within Cameroon. Incorporating feeds enriched with *Cetobacterium* or its beneficial metabolites could improve fish health, increase yields, and enhance economic returns for aquaculture operations. This highlights the importance of understanding the functional roles of key bacterial symbionts within fish microbiomes and leveraging this knowledge to optimize aquaculture practices for the benefit of both fish health and production efficiency.

The exploration of the bacteriome across fish gills, intestines, and pond water environments revealed varying abundances of *Cetobacterium* across these environments. Overall, *Cetobacterium* emerged as the most abundant bacterial genus in this study. In the gills, bacteria belonging to the phylum Fusobacteria constituted 49.95% of the phyla, with *Cetobacterium* accounting for 49.87% of all genera. Similarly, in the intestines, Fusobacteria were the most abundant phylum (65.3%), with *Cetobacterium* species accounting for 65.18% of all bacterial genera. However, such dominance of the Fusobacteria phyla was not reflected in pond water. Instead, Proteobacteria emerged as the most dominant phylum, accounting for 40.24% of all phyla identified. Bacteroidetes and Fusobacteria made up 24.28% and 24.12% of the phyla in pond water, respectively. Although *Cetobacterium species* remained the most abundant genus in pond water, they constituted a much lower proportion of the overall phyla and genera compared to gills and intestines. This suggests a distinct microbial composition and distribution across different aquatic environments, with *Cetobacterium* being particularly prevalent within the internal organs of fish than in the surrounding water. The differential abundance of *Cetobacterium* highlights its potential role as an important member of the fish microbiome, particularly in the gastrointestinal tract, and underscores the need for further investigation into its functional significance in different ecological contexts.

The current study identified prevalent bacteria genera, recognized as potential fish pathogens such as *Edwardsiella, Aeromonas, Plesiomonas,* and *Flavobacterium* across fish and pond water samples. This finding aligns with reports of gut-associated pathogenic microbiota in other freshwater aquaculture fish like tilapia [41]. Of these, *Edwardsiella* and *Aeromonas* species are recognized as emerging infectious bacterial pathogens [42, 43] in freshwater fish, while *Plesiomonas* are increasingly being recognized as an important pathogen to various fish [44]. *Flavobacterium* is also noted as a significant pathogen in catfish aquaculture [45, 46]. Investigations of bacterial communities in recirculating catfish aquaculture systems have similarly identified *Edwardsiella* and *Aeromonas* species, along with other key fish pathogenic species such as *Mycobacterium, Pseudomonas, Flavobacterium, Yersinia,* and *Rickettsia* in the aquaculture system [47]. Therefore, these pathogens pose significant concerns for fish health and aquaculture management, underscoring the importance of continued monitoring and mitigation efforts in aquaculture environments.

Shared bacteria in aquaculture systems included human gut bacteria like *Clostridium* and *Bacteroides*. *Clostridium* species include *Clostridium botulinum,* an important food-borne pathogen [48] among the most recurrent fish-related foodborne illnesses affecting humans because of their wide environmental distribution and spore-forming ability. Detailed investigations of the aquaculture ecosystems within this under-researched region are crucial for identifying major circulating fish pathogens, aiding effective disease surveillance and prevention.

In addition to *Cetobacterium*, which is the most prevalent bacteria genera with potential probiotic benefits for fish physiology [36], *Lactococcus* species have also been identified. Mainly classified as lactic acid bacteria (LAB), *Lactococcus* includes species such as *Lactococcus lactis* and *Lactococcus garvieae*. While *Lactococcus lactis* is a well-known probiotic bacterium, *Lactococcus garvieae* is recognized as a fish pathogen in marine and aquaculture environments [49, 50]. *Lactococcus lactis* has been studied as a probiotic for aquaculture, demonstrating its antimicrobial properties against *Lactococcus garvieae,* the causative agent of lactococcosis in fish [49, 50]. This therefore suggests the potential use of *Lactococcus lactis* as a probiotic agent in aquaculture for disease management.

The annotated functional profile of the bacteriome associated with catfish aquaculture revealed highly abundant KOs (K21572, K01190, K00059, and K03392) with potential involvement in the metabolism of carbohydrates, lipids, and amino acids (Figure 2). In addition to the potential probiotic species earlier highlighted, these functional profiles can offer insights that can be used to increase nutrient availability for cultured catfish with more locally available feed material [51]. Analysis also revealed the functional profile K00798, a cob(I)alamin adenosyltransferase that converts cobalamin (Vitamin B_12_), highly produced by *Cetobacterium,* to adenosylcobalamin, which is required as a cofactor for the activity of other enzymes [52]. Also important to highlight is the high expression of functional profiles (K02014 and K2157) with high affinity for binding iron under iron-limiting conditions. This can also serve to balance iron in the pond environment given that excess iron can cause stress in fish [53].

Analysis also revealed the presence of KO K09678, coding for a sulfotransferase expressed by sulfur-oxidizing bacteria that can be very useful in the metabolism and removal of hydrogen sulfide in an aquaculture environment [54]. The KO K03585, highly expressed in intestinal samples, demonstrates the presence of antimicrobial efflux pumps in the gut bacteriome of the African catfish. This presence can have implications for the use of antibiotics in aquaculture and the spread of AMR. Knowledge of the high expression KOs of the microbiome of catfish intestines and pond water sheds light on the composition of the gut microbiome in healthy catfish individuals. This insight can be used to design and develop strategies to promote catfish gut health.

The bacteriome of catfish gills, intestines, and pond water was predominantly composed of bacteria of the order Fusobacteriales and genus *Cetobacterium*. Additionally, pathogenic genera like *Edwardsiella*, *Aeromonas*, *Plesiomonas*, and *Flavobacterium,* along with human gut bacteria like *Clostridium and Bacteroides*, were identified. Furthermore, genera like *Lactococcus,* known for their probiotic properties, were also identified. These findings provide preliminary insight into the natural bacteriome of healthy catfish and pond water within the aquaculture environment in Cameroon. It underscores the potential role of freshwater fish aquaculture in harboring both human pathogenic bacteria and important probiotic species. Understanding the composition of these microbial communities and their delicate balance is crucial for managing fish health and ensuring the safety of aquaculture practices in Cameroon. Continued research is vital for optimizing aquaculture practices and ensuring food safety.

## Figures and Tables

**Table 1:** Taxonomic identification of samples represented by the percentage of total sequences. Included in this are genera accounting for ≥ 1% of sequences in at least one sample category. Shared taxa are present in all three samples. Unique genera are present in only one or two sample types.

	**Classification**					**Abundance (%)**		
	**Phylum**	**Class**	**Order**	**Family**	**Genus**	**Gills**	**Intestines**	**Pond water**
Shared taxa	Fusobacteria	Fusobacteria_c	Fusobacteriales	Fusobacteriaceae	*Cetobacterium*	54.35	64.39	26.29
	Bacteroidetes	Bacteroidia	Bacteroidales	Bacteroidaceae	*Bacteroides*	4.98	11.33	6.63
	Bacteroidetes	Bacteroidia	Bacteroidales	Porphyromonadaceae	GQ360021_g	4.56	5.01	2.87
	Proteobacteria	Gammaproteobacteria	Enterobacterales	Hafniaceae	*Edwardsiella*	4.37	2.82	1.17
	Firmicutes	Clostridia	Clostridiales	Clostridiaceae	*Clostridium*	0.83	3.5	2.5
	Proteobacteria	Gammaproteobacteria	Enterobacterales	Enterobacteriaceae	*Plesiomonas*	1.69	3.07	2.38
	Bacteroidetes	Bacteroidia	Bacteroidales	Bacteroidaceae	Bacteroidaceae_uc	0.12	3.1	0.62
	Proteobacteria	Betaproteobacteria	Rhodocyclales	Azovibrio_f	AF236014_g	3.17	0.52	1.84
	Bacteroidetes	Bacteroidia	Bacteroidales	Porphyromonadaceae	Porphyromonadaceae_uc	0.3	2.38	0.95
	Firmicutes	Bacilli	Lactobacillales	Streptococcaceae	*Lactococcus*	3.42	0.05	0.45
	Bacteroidetes	Flavobacteria	Flavobacteriales	Flavobacteriaceae	*Flavobacterium*	2.3	0.01	1.48
	Proteobacteria	Betaproteobacteria	Burkholderiales	Sutterellaceae	DQ196633_g	0.45	0.36	1.08
	Proteobacteria	Gammaproteobacteria	Aeromonadales	Aeromonadaceae	*Aeromonas*	1.26	0.02	0.31
	Proteobacteria	Gammaproteobacteria	Aeromonadales	Aeromonadaceae	*Pseudaeromonas*	0.39	0.01	1.14

Unique taxa	Proteobacteria	Betaproteobacteria	Burkholderiales	Comamonadaceae	*Sphaerotilus*	0.07	—	8.27
	Proteobacteria	Gammaproteobacteria	Cellvibrionales	Cellvibrionaceae	*Cellvibrio*	0.38	—	3.7
	Proteobacteria	Betaproteobacteria	Burkholderiales	Sutterellaceae	Sutterellaceae_uc	3.75	—	—
	Proteobacteria	Betaproteobacteria	Burkholderiales	Comamonadaceae	*Paucibacter*	1.02	—	2.46
	Proteobacteria	Betaproteobacteria	PAC001921_o	PAC001921_f	PAC001921_f_uc	3.58	—	—
	Bacteroidetes	Sphingobacteriia	Saprospirales	Saprospiraceae	EU104080_g	0.03	—	2.47
	Proteobacteria	Betaproteobacteria	Burkholderiales	Comamonadaceae	Comamonadaceae_uc	0.29	—	1.1
	Bacteroidetes	Cytophagia	Cytophagales	Cytophagaceae	Cytophagaceae_uc	0.02	—	1.01

**Table 2:** Principal functional profile of the catfish gills, intestines, and pond water microbiome.

**Orthology**	**Definition**	**p** ** value**	**Intestines**	**Gills**	**Pond water**
K02014	Iron complex outermembrane recepter protein	0.040	**0.427**	0.188	0.241
K21572	Starch-binding outer membrane protein, SusD/RagB family	0.040	**0.495**	0.130	0.177
K03088	RNA polymerase sigma-70 factor, ECF subfamily	0.043	**0.312**	0.127	0.203
K03585	Membrane fusion protein, multidrug efflux system	0.047	**0.166**	0.114	0.080
K04763	Integrase/recombinase XerD	0.047	**0.193**	0.104	0.069
K03654	ATP-dependent DNA helicase RecQ	0.047	**0.136**	0.093	0.065
K21573	TonB-dependent starch-binding outer membrane protein SusC	0.040	**0.319**	0.090	0.115
K03832	Periplasmic protein TonB	0.047	**0.117**	0.081	0.071
K07114	Ca-activated chloride channel homolog	0.047	**0.154**	0.077	0.069
K01190	Beta-galactosidase	0.050	**0.197**	0.076	0.073
K07720	Two-component system, response regulator YesN	0.047	**0.124**	0.070	0.063
K07407	Alpha-galactosidase	0.047	**0.119**	0.067	0.040
K01187	Alpha-glucosidase	0.047	**0.126**	0.060	0.041
K12340	Outer membrane protein	0.047	**0.117**	0.059	0.056
K07636	Two-component system, OmpR family, phosphate regulon sensor histidine kinase PhoR	0.050	**0.112**	0.058	0.064
K07165	Transmembrane sensor	0.047	**0.111**	0.038	0.055
K05349	Beta-glucosidase	0.040	**0.107**	0.031	0.045
K07760	Cyclin-dependent kinase	0.047	0.017	**0.099**	0.092
K03392	Aminocarboxymuconate-semialdehyde decarboxylase	0.047	0.038	0.286	**0.394**
K00059	3-Oxoacyl-[acyl-carrier protein] reductase	0.046	0.144	0.211	**0.306**
K02020	Molybdate transport system substrate-binding protein	0.040	0.047	0.098	**0.132**
K00798	Cob(I)alamin adenosyltransferase	0.047	0.041	0.083	**0.112**
K09678	[Heparan sulfate]-glucosamine 3-sulfotransferase 4	0.030	0.003	0.062	**0.204**
K12132	Eukaryotic-like serine/threonine-protein kinase	0.043	0.076	0.053	**0.117**
K00010	Myo-inositol 2-dehydrogenase/D-chiro-inositol 1-dehydrogenase	0.030	0.047	0.033	**0.114**

*Note:* Highest values are in bold.

## Data Availability

The datasets generated and used during the current study are presented in the article and the sequences deposited in the NCBI database under the Accession Number PRJNA1171439.

## References

[1] MinCommerce (2020). Fish From Local Production: The Government is Committed to Structuring the Sector. https://www.mincommerce.gov.cm/en/fish-local-production-government-committed-structuring-sector.

[2] Engwali F. D., Brenda B. D., Ewoukem E. T. (2019). Evaluation of the Economic Performance of Freshwater Fish Farming in the Wouri Division, Littoral Region of Cameroon. *International Journal of Fisheries and Aquatic Studies*.

[3] Mkong C. J., Molua E. L., Mvodo S. (2018). Determinants of Profitability of Fish Farming in Cameroon. *Agriculture, Forestry and Fisheries*.

[4] FAO (2022). *The State of World Fisheries and Aquaculture 2022: Towards Blue Transformation*.

[5] Santos L., Ramos F. (2018). Antimicrobial Resistance in Aquaculture: Current Knowledge and Alternatives to Tackle the Problem. *International Journal of Antimicrobial Agents*.

[6] Pulkkinen K., Suomalainen L.-R., Read A. F., Ebert D., Rintamäki P., Valtonen E. T. (2010). Intensive Fish Farming and the Evolution of Pathogen Virulence: The Case of Columnaris Disease in Finland. *Proceedings of the Royal Society B: Biological Sciences*.

[7] Sundberg L.-R., Ketola T., Laanto E. (2016). Intensive Aquaculture Selects for Increased Virulence and Interference Competition in Bacteria. *Proceedings of the Royal Society B: Biological Sciences*.

[8] Rekhari Y. C., Ritu A., Malobica D. T., Hema T. (2014). Qualitative and Quantitative Study on Bacterial Flora of Farm Raised Common Carp, Cyprinus carpio in India. *African Journal of Microbiology Research*.

[9] Fan L., Chen J., Meng S. (2017). Characterization of Microbial Communities in Intensive GIFT Tilapia (*Oreochromis niloticus*) Pond Systems During the Peak Period of Breeding. *Aquaculture Research*.

[10] Dinev T., Velichkova K., Stoyanova A., Sirakov I. (2023). Microbial Pathogens in Aquaponics Potentially Hazardous for Human Health. *Microorganisms*.

[11] Pakingking R., Palma P., Usero R. (2015). Quantitative and Qualitative Analyses of the Bacterial Microbiota of Tilapia (*Oreochromis niloticus*) Cultured in Earthen Ponds in the Philippines. *World Journal of Microbiology and Biotechnology*.

[12] Nicholson P., Fathi M. A., Fischer A. (2017). Detection of Tilapia Lake Virus in Egyptian Fish Farms Experiencing High Mortalities in 2015. *Journal of Fish Diseases*.

[13] Osman K. M., Al-Maary K. S., Mubarak A. S. (2017). Characterization and Susceptibility of Streptococci and Enterococci Isolated From Nile Tilapia (*Oreochromis niloticus*) Showing Septicaemia in Aquaculture and Wild Sites in Egypt. *BMC Veterinary Research*.

[14] Martins A. F. M., Pinheiro T. L., Imperatori A. (2019). Plesiomonas shigelloides: A Notable Carrier of Acquired Antimicrobial Resistance in Small Aquaculture Farms. *Aquaculture*.

[15] Nchanji G. T., Tchamba Kemajou A. L., Fossi B. T., Tchatat N. M., Ritter M., Wanji S. (2022). Site Specific Bacterial Load, Enterobacterial Occurrence and Antibiotic Susceptibility Patterns in Nile Tilapia (*Oreochromis niloticus*), Water and Sediment From Earthen Aquaculture Ponds. *PAMJ-One Health*.

[16] Newaj-Fyzul A., Mutani A., Ramsubhag A., Adesiyun A. (2008). Prevalence of Bacterial Pathogens and Their Anti-Microbial Resistance in Tilapia and Their Pond Water in Trinidad. *Zoonoses and Public Health*.

[17] Kaktcham P. M., Temgoua J.-B., Ngoufack Zambou F., Diaz-Ruiz G., Wacher C., Pérez-Chabela M. D. (2017). Quantitative Analyses of the Bacterial Microbiota of Rearing Environment, Tilapia and Common Carp Cultured in Earthen Ponds and Inhibitory Activity of Its Lactic Acid Bacteria on Fish Spoilage and Pathogenic Bacteria. *World Journal of Microbiology and Biotechnology*.

[18] Economou V., Gousia P. (2015). Agriculture and Food Animals as a Source of Antimicrobial-Resistant Bacteria. *Infection and Drug Resistance*.

[19] Burridge L., Weis J. S., Cabello F., Pizarro J., Bostick K. (2010). Chemical Use in Salmon Aquaculture: A Review of Current Practices and Possible Environmental Effects. *Aquaculture*.

[20] Chen J., Sun R., Pan C., Sun Y., Mai B., Li Q. X. (2020). Antibiotics and Food Safety in Aquaculture. *Journal of Agricultural and Food Chemistry*.

[21] Cabello F. C., Godfrey H. P., Buschmann A. H., Dölz H. J. (2016). Aquaculture as yet Another Environmental Gateway to the Development and Globalisation of Antimicrobial Resistance. *Lancet Infectious Diseases*.

[22] Meek R. W., Vyas H., Piddock L. J. V. (2015). Nonmedical Uses of Antibiotics: Time to Restrict Their Use?. *PLoS Biology*.

[23] Mugimba K. K., Chengula A. A., Wamala S. (2018). Detection of Tilapia Lake Virus (TiLV) Infection by PCR in Farmed and Wild Nile Tilapia (*Oreochromis niloticus*) From Lake Victoria. *Journal of Fish Diseases*.

[24] Ramírez-Paredes J. G., Paley R. K., Hunt W. (2021). First Detection of Infectious Spleen and Kidney Necrosis Virus (ISKNV) Associated With Massive Mortalities in Farmed Tilapia in Africa. *Transboundary and Emerging Diseases*.

[25] Done H. Y., Venkatesan A. K., Halden R. U. (2015). Does the Recent Growth of Aquaculture Create Antibiotic Resistance Threats Different From Those Associated With Land Animal Production in Agriculture?. *AAPS Journal*.

[26] WHO (2020). *Stop Using Antibiotics in Healthy Animals to Preserve Their Effectiveness*.

[27] Ragasa C., Charo-Karisa H., Rurangwa E., Tran N., Shikuku K. M. (2022). Sustainable Aquaculture Development in Sub-Saharan Africa. *Nature Food*.

[28] Kaktcham P. M., Zambou Ngoufack F., Fonteh Anyangwe F., Pérez-Chabela M. D., University of Dschang, Camerun (2015). Aquaculture in Cameroon and Potential of Lactic Acid Bacteria to Be Used as Diseases Controlling Agents. A Review. *Nacameh*.

[29] Skvortsova E., Filinskaya O., Postrash I., Bushkareva A., Mostofina A. (2023). Biodiversity of Gut Microorganisms in Aquacultured African Catfish. *E3S Web of Conferences*.

[30] Brumfield K. D., Raupp M. J., Haji D. (2022). Gut Microbiome Insights From 16S rRNA Analysis of 17-Year Periodical Cicadas (*Hemiptera*: *Magicicada* spp.) Broods II, VI, and X. *Scientific Reports*.

[31] García G., Martínez D., Soto J. (2023). Short-Chain Fructooligosaccharides Improve Gut Microbiota Composition in Patients With Type 2 Diabetes. A Randomized, Open-Label, Controlled Pilot Clinical Trial. *Journal of Biotechnology and Biomedicine*.

[32] Yoon S.-H., Ha S.-M., Kwon S. (2017). Introducing EzBioCloud: A Taxonomically United Database of 16S rRNA Gene Sequences and Whole-Genome Assemblies. *International Journal of Systematic and Evolutionary Microbiology*.

[33] Larsen A. M., Mohammed H. H., Arias C. R. (2014). Characterization of the Gut Microbiota of Three Commercially Valuable Warmwater Fish Species. *Journal of Applied Microbiology*.

[34] Ramírez C., Coronado J., Silva A., Romero J. (2018). Cetobacterium Is a Major Component of the Microbiome of Giant Amazonian Fish (*Arapaima gigas*) in Ecuador. *Animals*.

[35] Zhang Z., Fan Z., Yi M. (2022). Characterization of the Core Gut Microbiota of Nile Tilapia (*Oreochromis niloticus*): Indication of a Putative Novel Cetobacterium Species and Analysis of Its Potential Function on Nutrition. *Archives of Microbiology*.

[36] Yajima D., Fujita H., Hayashi I., Shima G., Suzuki K., Toju H. (2023). Core Species and Interactions Prominent in Fish-Associated Microbiome Dynamics. *Microbiome*.

[37] Elsaied H. E., Soliman T., Abu-Taleb H. T., Goto H., Jenke-Kodam H. (2019). Phylogenetic Characterization of Eukaryotic and Prokaryotic Gut Flora of Nile Tilapia, Oreochromis niloticus, Along Niches of Lake Nasser, Egypt, Based on rRNA Gene High-Throughput Sequences. *Ecological Genetics and Genomics*.

[38] Bereded N. K., Curto M., Domig K. J. (2020). Metabarcoding Analyses of Gut Microbiota of Nile Tilapia (*Oreochromis niloticus*) From Lake Awassa and Lake Chamo, Ethiopia. *Microorganisms*.

[39] Sugita H., Shibuya K., Shimooka H., Deguchi Y. (1996). Antibacterial Abilities of Intestinal Bacteria in Freshwater Cultured Fish. *Aquaculture*.

[40] Qi X., Zhang Y., Zhang Y. (2023). Vitamin B12 Produced by Cetobacterium somerae Improves Host Resistance Against Pathogen Infection Through Strengthening the Interactions Within Gut Microbiota. *Microbiome*.

[41] Debnath S. C., McMurtrie J., Temperton B., Delamare-Deboutteville J., Mohan C. V., Tyler C. R. (2023). Tilapia Aquaculture, Emerging Diseases, and the Roles of the Skin Microbiomes in Health and Disease. *Aquaculture International*.

[42] Nhinh D. T., Giang N. T. H., Mohamad K. V., Dang L. T., Dong H. T., Hoai T. D. (2022). Widespread Presence of a Highly Virulent *Edwardsiella ictaluri* Strain in Farmed tilapia, *Oreochromis* spp. *Transboundary and Emerging Diseases*.

[43] Monir S., Yusoff S. M., Mohamad A., Ina-Salwany M. Y. (2020). Vaccination of Tilapia Against Motile *Aeromonas* Septicemia: A Review. *Journal of Aquatic Animal Health*.

[44] Cortés-Sánchez A. D. J., Espinosa-Chaurand L. D., Díaz-Ramirez M., Torres-Ochoa E. (2021). Plesiomonas: A Review on Food Safety, Fish-Borne Diseases, and Tilapia. *Scientific World Journal*.

[45] Wise A. L., LaFrentz B. R., Kelly A. M. (2021). A Review of Bacterial Co-Infections in Farmed Catfish: Components, Diagnostics, and Treatment Directions. *Animals*.

[46] Akinola O. (2023). Pathogenicity and Virulence of Flavobacterium *columnare* Isolated From Farmed African Catfish, *Clarias gariepinus* (Burchell 1822). *Alexandria Journal of Veterinary Sciences*.

[47] Clols-Fuentes J., Nguinkal J. A., Unger P., Kreikemeyer B., Palm H. W. (2023). Bacterial Community in African Catfish (*Clarias gariepinus*) Recirculating Aquaculture Systems Under Different Stocking Densities. *Science*.

[48] Novoslavskij A., Terentjeva M., Eizenberga I., Valciņa O., Bartkevičs V., Bērziņš A. (2016). Major Foodborne Pathogens in Fish and Fish Products: A Review. *Annals of Microbiology*.

[49] Sequeiros C., Garcés M. E., Vallejo M., Marguet E. R., Olivera N. L. (2015). Potential Aquaculture Probiont *Lactococcus lactis* TW34 Produces Nisin Z and Inhibits the Fish Pathogen Lactococcus garvieae. *Archives of Microbiology*.

[50] Feito J., Araújo C., Arbulu S. (2023). Design of *Lactococcus lactis* Strains Producing Garvicin A and/or Garvicin Q, Either Alone or Together with Nisin A or Nisin Z and High Antimicrobial Activity Against *Lactococcus garvieae*. *Food*.

[51] Miégoué E., Zebaze P. D. T., Tendonkeng F. (2018). Effect of Replacement of Fishmeal With Lima Bean Meal on the Zootechnical Performances of African Catfish (Clarias Gariepinus) in the Batié Sub-Division, West Region of Cameroun. *International Journal of Aquaculture Research and Development*.

[52] Marsh E. N. G., Meléndez G. D. R. (2012). Adenosylcobalamin Enzymes: Theory and Experiment Begin to Converge. *Biochimica et Biophysica Acta*.

[53] Farooq A., Verma A. K., Hittinahalli C. M., Varghese T., Pathak M. S. (2023). Iron Supplementation in Aquaculture Wastewater and Its Impact on Osmoregulatory, Haematological, Blood Biochemical, and Stress Responses of Pangasius With Spinach in Nutrient Film Technique Based Aquaponics. *Aquaculture*.

[54] Dashtbin R., Mahmoudi N., Besharati H., Lalevic B. (2023). Identification of Sulfur-Oxidizing Bacteria From Fishponds and Their Performance to Remove Hydrogen Sulfide Under Aquarium Conditions. *Brazilian Journal of Microbiology*.

